# Neurobehavior of newborn infants exposed prenatally to methadone and identification of a neurobehavioral profile linked to poorer neurodevelopmental outcomes at age 24 months

**DOI:** 10.1371/journal.pone.0240905

**Published:** 2020-10-16

**Authors:** Trecia A. Wouldes, Lianne J. Woodward

**Affiliations:** 1 Department of Psychological Medicine, Faculty of Medical and Health Sciences, The University of Auckland, Auckland, New Zealand; 2 School of Health Sciences, University of Canterbury, Christchurch, New Zealand; Wayne State University, UNITED STATES

## Abstract

The abuse of prescription opioids and heroin by women of childbearing age over the past decade has resulted in a five-fold increase in the number of infants born opioid-dependent. Daily opioid substitution treatment with methadone is associated with less maternal illicit opioid use and improved antenatal care. However, research on the neurobehavioral effects of daily prenatal exposure to methadone on the infant is limited. Using the NICU Network Neurobehavioral Scale (NNNS), we compared the neurobehavior at birth of 86 infants born to opioid-dependent mothers receiving methadone treatment (MMT) with 103 infants unexposed to methadone. Generalized linear models, adjusted for covariates, showed methadone exposed infants had significantly poorer attention, regulation, and quality of movement. They were also significantly more excitable, more easily aroused, exhibited more non-optimal reflexes, hypertonicity, and total signs of stress abstinence. Maternal MMT was also associated with more indices of neonatal abstinence, including: CNS, visual, genitourinary (GI), and state. Latent profile analysis of the NNNS summary scores revealed four distinct neurobehavioral profiles with infants characterized by the most disturbed neurobehavior at birth having the poorest clinical outcomes at birth, and poorer cognitive and motor development at 24 months of age.

## Introduction

Globally, illicit opioids are the most harmful drug to human health [1]. Opioid use is associated with an increased risk of fatal and non-fatal overdoses, as well as infectious diseases such as HIV and Hepatitis C. As a result, opioids account for 12 million disability adjusted life years or 70% of the global burden of drug-related disabilities and deaths. This trend is most prominent in the United States, where opioid-related deaths increased threefold between 1999 and 2015 [1, 2].

Alongside these global trends, there has been a corresponding increase in the use and abuse of prescription opioids, and more recently, heroin, in women of childbearing age [2–4]. This has, in turn, given rise to a three-fold increase in the rate of neonatal abstinence syndrome (NAS) from 2000 to 2009, increasing to a five-fold increase in NAS or neonatal opioid withdrawal syndrome (NOWS) between 2009 and 2012 [5–12]. Typically, the signs of opioid withdrawal include evidence of central nervous system (CNS) irritability, gastrointestinal dysfunction, yawning, sneezing, and fever. Behaviorally, opioid abstinent babies frantically suck their fingers, display incessant and inconsolable high-pitched crying, and are restless and irritable [5–9]. Most of these signs of withdrawal will disappear over the first few months of postnatal life, but CNS disturbance can persist for up to 4 to 6 months [9]. As a result, a significant number of these infants may require extended neonatal intensive care and pharmacotherapy after birth [13].

Daily opioid substitution therapy during pregnancy with either methadone (MMT) or buprenorphine (BUP) has been shown to reduce maternal illicit opioid use, improve prenatal care, and reduce maternal symptoms of craving and withdrawal [14–17]. Lifestyle improvements and reductions in illegal activity are also seen [18]. However, high rates of neonatal abstinence (40% to 80%) are still observed in both MMT and BUP exposed infants [14, 15, 19, 20]. Methadone is now the most commonly reported maternal medication associated with NAS (31%), followed by opioid pain relievers (24%) and buprenorphine (15%) [13]. Despite emerging evidence suggesting that buprenorphine substitution therapy may result in less severe and less prolonged NAS, in most centers, MMT remains the standard of care for treating opioid dependence during pregnancy [14, 21–23].

Infant adaptation to the postnatal environment is important for establishing optimal patterns of sleep, feeding, growth and in promoting parent-infant connection [24]. Early neurobehavioral studies using the Brazelton Neonatal Behavioral Assessment Scale (NBAS) showed that infants exposed prenatally to MMT were more state labile and exhibited more tremors, crying, hypertonicity, and behavioral dysregulation than unexposed infants [25]. More recent studies have compared the neurobehavior of methadone and buprenorphine exposed infants [21, 26–30] as well as those treated for NAS or not [31, 32]. These studies used the Neonatal Intensive Care Unit Network Neurobehavioral Scale (NNNS) [33], which is a well-validated neurobehavioral measure designed specifically for the drug-exposed infant. Based on the NBAS, the NNNS assesses neurological and behavioral functioning and the extent of stress abstinence [33]. For example, a comparison of 21 MMT exposed and 16 BUP exposed infants found that MMT infants were more easily aroused, showed more signs of stress abstinence, and were more excitable [29].

Two further studies compared MMT exposed infants requiring pharmacotherapy for NAS and those who did not [31, 32]. One found that infants who received pharmacotherapy to treat their NAS were less able to habituate to visual and aural stimuli, were more easily aroused and excitable, and displayed more hypertonicity than untreated infants [31]. When the NNNS scores of both treated and untreated study infants were compared to normative data from an unexposed group [34], significant differences were found in scores of habituation, attention, handling, non-optimal reflexes, hypertonicity, hypotonicity and stress abstinence. In contrast, the second study found no neurobehavioral differences at 6 weeks postpartum between those receiving pharmacotherapy for NAS and those who did not [32]. However, both treated and untreated infants were characterized by more stress abstinence signs than unexposed comparison infants. Treated infants also had poorer self-regulation and quality of movements than comparison infants [32].

An enduring question concerning children exposed prenatally to MMT is whether there are ongoing effects on neurodevelopment. However, there is a lack of well-controlled prospective longitudinal studies comparing the neurodevelopment of MMT exposed and unexposed infants. Of the 16 studies carried out prior to 1990 where comparative longitudinal data were reported, only five found significant, but small differences between MMT exposed and unexposed infants on the Mental Development Index (MDI) [35–39], and four found differences on the Psychomotor Development Index (PDI) [36, 38, 40, 41] of the Bayley Scales of Infant Development II [42]. Notable were the small samples in these studies and the low mean daily methadone dose (14–40 mg/day) prescribed to mothers at that time. The influence of other factors that might contribute to between group differences was also rarely adequately taken into account. More recently, the escalation in prescription opioid abuse and heroin has resulted in over 50 published reports on the neurodevelopment of children exposed prenatally to opioids, but few are prospective, well-controlled longitudinal studies of MMT (see review by Conradt et al. [43]). Of those prospective studies that compared children exposed prenatally to MMT with unexposed children, findings are equivocal with one study finding no differences in early cognitive or motor development [44], one study finding mild impairment in cognitive development [45], one lower scores on psychomotor outcomes at 6 months [46], and two other studies finding lower scores on cognitive outcomes at ages 4 [47], and 4.5 and 5.5 [48] years. However, most of these studies were limited by the inclusion of small numbers of participants or did not control for other drug use, which may explain the inconsistent findings.

A further explanation may be the varying infant susceptibility to the effects of prenatal exposure to MMT and other maternal and environmental factors that result in poorer neurodevelopmental outcomes for some groups of children. To date, most studies that have examined the effects of maternal MMT on the neurobehavior of the infant have used a variable-centered analysis, such as linear or multiple regression which examines the relationship between variables (e.g., to predict outcomes). These analyses assume participants have been sampled from a single population and do not take into account the unobserved heterogeneity that may exist within that population; they, therefore cannot explore how the heterogeneity in neurobehavioural profiles may differentially relate to MMT-related outcomes [49]. In comparison, person-centered analyses assume heterogeneity within the sampled population and that important subgroups of individuals can be identified by shared neurobehavioral attributes. Latent profile analyses, a person-centered approach, uses observed continuous data to assign class or group membership [50]. In this procedure, instead of focusing on a single component of neurobehaviour, latent profile analyses can use the full pattern of co-occurring neurobehavioral responses to categorize subgroups of infants. For instance, employing a person-centered analysis of the summary scores of the NNNS data, subgroups, or profiles of infants have been identified that are linked to poorer medical and behavioural outcomes. In a sample of infants exposed prenatally to cocaine, opiates, and other psychoactive drugs [51], distinct profiles of the NNNS summary scores obtained using latent profile analysis were associated with poorer clinical outcomes at birth and neurodevelopmental consequences that persisted over the first 4.5 years.

Given steep increases in NICU referrals of infants exposed prenatally to methadone and other opioids, the lack of well-controlled, longitudinal outcome studies, existing contradictory neurodevelopmental data, and recent findings showing evidence of neurobehavioral dysregulation among infants exposed to MMT, there is a clear need to better understand the early neurobehavior of these infants. Also important is the identification of those infants who may be more at risk for adverse neurodevelopmental outcomes at birth that extend beyond the perinatal period.

Using data from the prospective, longitudinal Christchurch Methadone In Pregnancy (MIP) Study, this report aims to investigate the following. First, to examine the neurobehavior of MMT exposed infants at term equivalent relative to an unexposed group of infants on the NNNS. Second, to use a latent profile analysis of the summary scores on the NNNS to determine if there is a distinct profile of neurobehavior that characterizes infants with poorer clinical outcomes at birth and at 24 months.

## Methods

### Participants

The study sample included two groups of infants whose mothers were recruited during their second or third trimester of pregnancy in Christchurch, New Zealand, from 2003 to 2008.

#### Methadone exposed group

The first study group consisted of a consecutive series of 100 infants born to opioid-dependent women receiving daily methadone maintenance treatment (MMT) through the only regional service providing opioid substitution therapy. Inclusion criteria required mothers to be enrolled in MMT prior to their third trimester. Exclusion criteria included mother unable to give informed consent, poor spoken English, intention to deliver outside the region, very preterm birth (≤ 32 weeks gestation), fetal alcohol syndrome, infant congenital abnormalities or HIV positive. Over the recruitment period, 119 women were eligible for inclusion in the MMT group. Of these, 99 were recruited (83%), resulting in 100 live births (58 male, 42 female), including one set of twins. Reasons for sample loss included failure to recruit (n = 2) and refusal (n = 17). NNNS data for a further 8 infants were incomplete or missing, with an additional 6 infants needing to be excluded due to untestability on the NNNS defined as five unsuccessful assessment attempts.

Three quarters (76%) of mothers in the MMT group were receiving opioid substitution therapy when they became pregnant. Pregnancy doses ranged from 12.5 to 195.0 mg/day, with the highest mean methadone dose per trimester being 73.1±31.1mg/day in trimester 1, 67.8+31.8mg/day in trimester 2, and 68.9±34.3mg/day in trimester 3. A higher proportion of mothers increased their dose (33% 2^nd^ semester and 21% 3^rd^ semester) than decreased their dose (15% and 16%) over their pregnancy. The average maternal methadone dose across pregnancy was 61.8±35.3mg/day.

#### Unexposed comparison group

The second study group consisted of 110 infants born to women who were not enrolled in MMT. These infants were randomly selected from the maternity booking schedule for the Canterbury District Health Board, which serves both rural and urban areas within the Canterbury region. The same exclusion criteria were applied. A total of 169 untreated comparison women were identified. Of these, 108 women were recruited (65%), resulting in 110 live births (48 male, 62 female), including two sets of twins. Reasons for non-recruitment included inability to trace (*N* = 20) and refusal (*N* = 39). Control infants were born during the same time period and matched for expected birth date with children in the MMT group. Comparison of the socio-economic profile of women in this group with regional census data (Statistics NZ 2006) showed that this cohort was representative of the region from which they were recruited. Table 1 describes the maternal and infant characteristics of participants included in this report.

**Table 1:** Maternal background, obstetric history, and drug use during pregnancy and infant clinical characteristics at birth.

	Exposed MMT *N* = 86	Comparison *N* = 103	*P* value
**Maternal Demographics & Background**			
Maternal age (yr)	30.07 (6.11)	31.07 (6.61)	.289
NZ Maori	16 (19%)	12 (12%)	.178
NZ European	71 (83%)	88 (85%)	.292
Pacific Islands, Asian & other	2 (2%)	8 (8%)	.097
Single parent family	34 (40%)	18 (18%)	.001
No maternal educational qualification	56 (65%)	34 (33%)	< .001
Social welfare dependent	79 (93%)	18 (18%)	< .001
**Maternal Factors**			
Primigravida	6 (7%)	39 (38%)	< .001
History of termination	36 (42%)	12 (12%)	< .001
Previous live births	2 (0–7)	1 (0–7)	< .001
Any maternal or hospital report of mental illness	55 (64%)	14 (14%)	< .001
**Maternal Pregnancy Drug Use**			
Alcohol use in pregnancy	14 (16%)	22 (21%)	.376
Average drinks per week	0.75 (2.71)	0.33 (1.04)	.146
Median (range)	0 (0–20)	0 (0–8)	
Smoked cigarettes in pregnancy	80 (93%)	17 (17%)	< .001
Average cigarettes per day	13.64 (9.27)	1.42 (3.92)	< .001
Median (range)	11 (0–40)	0 (0–15)	
Marijuana	45 (52%)	2 (2%)	< .001
Average joints per week	1.28 (2.20)	0.03 (0.10)	< .001
Median (range)	0.33 (0–10)	0.0 (0–1)	
Benzodiazepines	23 (27%)	0 (0%)	< .001
Average times used per week	0.71 (1.99)	0	< .001
Median (range)	0 (0–14)	0	
Stimulants	16 (19%)	1 (%)	< .001
Average times used per week	0.58 (1.62)	0.03 (0.26)	.001
Median (range)	0 (0–9)	0 (0–3)	
Illicit opiates	22 (26%)	0 (0%)	< .001
Average times used per week	1.27 (2.99)	0	< .001
Median (range)	0 (0–19)	0	
Prescribed medications	24 (28%)	17 (17%)	.058
Ritalin	5 (5.8%)	0	.018
SSRIs	2 (2.3%)	5 (4.9%)	.458
Benzodiazepines	4 (4.7%)	1 (1.0%)	.179
Used > 2 prescribed, legal or illegal drugs	35 (41%)	2 (2%)	< .001
**Infant Birth Outcomes**			
Male	49 (57%)	47 (46%)	.120
Apgar 1	8.21 (1.72)	8.32 (1.55)	.657
Apgar 5	9.63 (0.66)	9.52 (0.93)	.375
Born preterm < 37 weeks	11 (13%)	7 (7%)	.162
Gestational age (weeks)	38.76 (1.80)	39.25 (1.69)	.054
Birth weight (gm)	3049.73 (459.74)	3428.03 (587.15)	< .001
Birth head circumference (cms)	33.88 (1.51)	34.73 (1.44)	< .001
Birth length (cm)	50.17 (3.10)	52.18 (3.15)	< .001
Referred to NICU	13 (16%)	2 (2%)	.001
Days in Special Care Baby Unit	11 (0–55)	0 (0–14)	< .001
Total days in hospital	12 (0–55)	3 (0–14	< .001
Corrected gestational age NNNS completed	41.93 (2.19)	41.83 (1.44)	.713
Treated for NAS	73 (88%)	-	-
Treated with morphine only	55 (64%)		
Treated with morphine & phenobarbital	18 (22%)		

Data are Mean (SD), Median (range) or *N* (%). Maternal drug use is reported in *N* (%), average cigarettes per day, drinks per week, joints per week, and average number of times other opiates, stimulants, and benzodiazepines used per week across pregnancy, median, and range.

### Procedure

Ethics approval was obtained from the Canterbury Regional Ethics Committee, and written informed consent obtained from all mothers. Near the end of the third trimester or directly after birth, mothers were interviewed about their socioeconomic status, education, and family circumstances as well as their obstetric, mental health and alcohol, tobacco, prescription and illicit drug use history (Table 1). This interview was conducted by a senior research nurse who was aware of the group status of the mothers but did not know the dose of methadone mothers in the MMT group were prescribed or the length of time they had been receiving MMT. Maternal and infant chart reviews provided further information about maternal obstetric history, mental health status, prescribed medications that included antidepressants, antipsychotics, and anti-epileptics, and infant clinical outcomes. A detailed description of the psychosocial characteristics and patterns of poly-drug use of women enrolled in the MIP study is published elsewhere [52].

### Measures

#### Maternal licit and illicit drug use

Detailed information about maternal use of licit and illicit psychoactive drugs and dependence during pregnancy was obtained from the Composite International Diagnostic Interview (CIDI) [53]. For each substance (tobacco, alcohol, cannabis, opiates, benzodiazepines, and stimulants), women were asked about the frequency and extent of their use before pregnancy and during each trimester. For the purposes of these analyses we used the average number of cigarettes per day, the average number of drinks consumed and joints smoked per week, and the average number of times stimulants, other opiates and benzodiazepines were used per week across pregnancy. Finally, maternal licit and illict psychoactive drug use was further independently assessed via random maternal urine samples during pregnancy of 49 (61%) of MMT women (2.8 samples/woman). This information was cross-checked against maternal self-reported drug use to create overall measures of maternal pregnancy licit and illicit drug use. Discrepant positive urine results were found for only 5 of the 49 women who had not reported use of a specific substance. For analysis, women who reported use of a licit or illicit psychoactive drug or who had a positive urine test result of one were considered to have used that drug during the trimester in which the test was carried out.

#### NICU Network Neurobehavioral Scale (NNNS)

All study infants had an NNNS assessment within one week of birth or corrected gestational age for infants born preterm (32–37 weeks) after they were deemed stable by the medical team. The median and range of ages at assessment for each group were: MIPS median age = 42.00, range 37.00 to 49.00 vs Comparison median age = 42.00, 39.00 to 46.00. For most infants, this was done prior to hospital discharge at a time when they were midway between feeds, and the infant was asleep. For a small proportion of infants discharged early from the hospital (*N* = 12, 6%), to minimize sample loss, the NNNS was done at home in a quiet room under the same conditions. The NNNS is a standardized neurological exam which measures a newborn infant’s neurological function, behavior and signs of stress abstinence [33, 54]. “Packages” of neurologic behavior are administered in a set order to produce 13 summary scores based on structured observations of active and passive tone, primitive reflexes, state transitions, and orientation to animate and inanimate visual and aural stimuli and signs of stress/abstinence (Table 2). Higher scores on the attention, regulation, and quality of movement scales are more optimal. Whereas, lower scores on habituation, arousal, handling, excitability, lethargy, non-optimal and asymmetric reflexes, hypertonicity, and hypotonicity indicate better function. Stress/abstinence is scored as “yes” or “no” from a list of signs of withdrawal organized by organ system (50 items). The NNNS was administered by one of three examiners trained to reliability and blind to group status.

**Table 2:** Summary scores (excluding habituation) of the NICU Network Neurobehavioral Scale (NNNS).

Attention	High scores indicate an infant’s sustained ability to attend and respond appropriately to auditory and visual stimulation.
Handling	High scores suggest that the infant required substantial soothing and settling by the examiner to maintain a quiet alert state to elicit attention and to respond to auditory and visual stimuli.
Self-regulation	High scores suggest the infant has a better ability to self-regulate and cope with the demands of the NNNS examination.
Arousal	High scores suggest the infant exhibited higher levels of fussing, crying and associated motor activity throughout the examination.
Excitability	High scores indicate an infant exhibited high levels of motor, state, and physiological reactivity and irritability even with repeated attempts at soothing by the examiner.
Lethargy	High scores suggest the infant was continually under aroused.
Hypertonicity	High scores indicate the infant exhibited tight muscle tone in the arms, legs, and trunk.
Hypotonicity	High scores indicate the infant had consistently low muscle tone in the arms, legs and trunk.
Non-optimal Reflexes	The number of Non-optimal reflex responses on upper and lower extremities. (Higher scores less optimal).
Asymmetric Reflexes	The number of times a reflex on one side of the body is stronger/weaker than the other. (Higher scores less optimal)
Quality of Movement	High scores suggest more mature motor control, including: smoothness and modulation of movement of the arms and legs, as well as tremors and startles.
Stress Abstinence	The number of stress abstinence signs an infant exhibited across seven categories: Autonomic, physiological, CNS, visual, gastrointestinal, skin, state. (High scores less optimal)

#### Neurodevelopment at age 24 months

The Bayley Scales of Infant Development Version 2 (BSID-II) [55] was administered by a blinded clinical psychologist at age 2 years (corrected for prematurity) as part of a larger neurodevelopmental follow-up. The BSID-II is a standardized measure of child cognitive (Mental Developmental Index or MDI) and neuromotor function (Psychomotor Developmental Index or PDI).

### Statistical analysis

#### Exploratory data analysis

All analyses were conducted with SPSS version 26. All infant birth data were complete, with 96% of the infants available for follow-up at 24 months on the Bayley MDI and PDI. Outliers were identified by visually examining box plots of the methadone and comparison groups separately and combined. Outliers in the NNNS Summary scores were identified in both the MMT and comparison groups and were in the same direction (either higher or lower scores) for Non-optimal reflexes, Attention, Handling, Stress Abstinence, Quality of Movement, Asymmetrical Reflexes. Outliers were also found for both groups in the summary scores of physiological, autonomic, visual, and Genitourinary (GI) stress. Analyses were run with and without outliers, and no substantial differences were found for any of the above summary scores. Assumptions of normality, linearity, and homogeneity of variance were confirmed with visual examination of the frequency distributions, histograms, and residual scatter plots. Skewness and kurtosis for all but four of the variables above were all around +/- 1.00. In addition, when the means and medians of these variables were compared, they were similar. Further visual examination of frequency distributions and histograms of Hypertonicity, Hypotonicity, Asymmetrical Reflexes and Excitability all showed distributions skewed towards more optimal summary scores (low scores) for all four scales for both groups, the subcategories of abstinence were also significantly skewed towards lower values.

Analyses were run with and without the 3 sets of twins included in our sample (1 set in the MMT group and 2 in the comparison group). As there were no differences when these were included or excluded in all analyses, results are reported for the whole sample.

#### Between group differences

*T*-tests, Kruskall-Wallis, and *Chi*-square statistics were used to examine social background, maternal licit and illicit psychoactive and prescribed drugs and other infant clinical differences at birth between MMT exposed and comparison groups at birth (Table 1). Robust empirical variance structures were used in generalized linear models (GLIM) to overcome slight deviations from normality and for those summary scores that were significantly skewed. Differences between the MMT exposed and comparison infants on individual summary scores of the NNNS and neurodevelopmental outcomes at age 24 months were tested using GLIM) with a link function. For those summary scores that were significantly skewed towards lower scores, gamma models with a log function were employed. GLIM were also used to compare the daily dose of methadone mothers were receiving during pregnancy and summary scores on the NNNS. MMT dose was categorized as no, low (≤64 mg) or high dose (≥65 mg) averaged across pregnancy, and similarly categorized for the highest individual recorded dose during the third trimester. High, low and no dose were dummy coded as 2, 1 and 0, respectively. High dose was selected as the reference category in GLIM models. GLIM analyses were conducted with and without covariates, as described below.

#### Adjustment for covariates

A range of covariates were selected based on previous research and theory linking these factors to pregnancy drug use and infant neurobehavioral outcomes (see Table 1). These were further checked for multicollinearity and composite measures created if necessary. GLIM models were then fitted using forwards and backwards variable elimination to identify the best fitting and most parsimonious models. Covariates were retained in the model if they changed the effect estimates of the unadjusted relation between NNNS summary scores and MMT exposure by more than 5%. All analyses were adjusted for the corrected age of NNNS assessment, socioeconomic status as well as the following covariates: 1) maternal prescription drug and psychoactive licit and illicit drug use during pregnancy including medications prescribed across pregnancy (SSRIs, benzodiazepines & ritalin), average number of cigarettes per day, average number of drinks and average number of joints of marijuana per week, average times benzodiazepines, other opiates or stimulants were used per week across pregnancy; 2) infant characteristics including birth weight, sex and gestational age in completed weeks; 3) maternal obstetric and mental health history including number of terminations, gravida and documented or self-reported history of mental illness.

#### Latent profile analysis

Latent profile analysis (LPA) was used to classify individual infants into homogeneous subgroups or latent profiles. Data suitable for LPA usually consists of unobservable subgroups of individuals with different probability distributions. LPA is an extension of Latent Class Analysis, a model-based clustering technique [56]. The advantage of LPA over LCA is that it can accommodate continuous, ordinal, and binary indicators. Profiles of infants in this study were determined using the 12 NNNS summary scores. Habituation summary scores were excluded since only 51% of the MMT exposed, and 37% of comparison infants completed this package. Hypertonicity and hypotonicity summary scores were dichotomized (yes/no), with all other scores treated as continuous variables. Infants who shared similar patterns across these scores were allocated to discrete profiles with the goal of minimizing the heterogeneity of the NNNS summary scores in one profile and maximizing the heterogeneity of scores across other profiles. Latent profile analyses were conducted with the M*plus* 7 statistical package using finite mixture modeling [57]. Random starts were used to ensure replication of the best log-likelihood and to avoid local maxima.

The final number of profiles was determined by exploring a varying number and selecting the profiles that made the most sense in relation to theory, and previous research [51, 58], the nature of the groups, and interpretation of the results—as well as alternate goodness-of-fit indices and tests of statistical significance (Table 3). Two profiles were initially specified and were increased by one in subsequent analyses until the final model was obtained. At each step, changes in the Bayesian Information Criterion (BIC) adjusted for sample size was used to assess model fit. The number of profiles was determined to be appropriate at the point where no significant drop in BIC was observed as the profile number increased. Model fit and optimum number of profiles were also assessed with the bootstrap log likelihood ratio (LR) difference test [59], and the average posterior probability (entropy).

**Table 3:** Model fit and model comparisons for 2, 3 or 4 profiles.

Latent Profiles	Sample-Size Adjusted Bayesian Information Criterion (BIC)^a^	Entropy^b^	Bootstrapped Log Likelihood Ratio Test for 1 Less Class or Profile^c^
2	4546.199	.932	-2484.238*
3	4457.749	.914	-2236.801*
4	4327.245	.911	-2153.002*

*p< .0001.

^a^Model fit lower values are better.

^b^Measure of how clearly distinguishable the classes are based on individual’s estimated class probability. This should be close to 1.0.

^c^Compares model with G profiles or classes with G– 1 (3 vs 4 profiles). A significant Ratio indicates the model with 1 more profile has a better relative fit than the model with 1 less profile (4 profiles better than 3).

When the final number of profiles were obtained, standardized scores were computed to compare summary scores on the same scale across the different profiles. Each summary score was subtracted from the overall mean of individual scores and divided by the overall standard deviation for that summary score. Differences in mean summary profiles on maternal and infant characteristics at birth, and neurodevelopmental outcomes at 24 months were examined using *t*-tests or Kruskall-Wallis for continuous variables and *Chi*-square for dichotomous variables.

## Results

### Neurobehavioral outcomes of infants exposed to methadone during pregnancy

There was no difference in the mean infant gestational age at the time of NNNS assessment between infants prenatally exposed to MMT and unexposed infants (MMT exposed M = 41.5±5.3 weeks vs unexposed M = 41.7±1.5 weeks). However, infants in the MMT group were significantly more likely to require more than 2 attempts to complete the NNNS than comparison group infants (11% v. 2%, *p* = .025). Twelve infants were administered the NNNS in the home after discharge from hospital (Mean gestational age at hospital = 39.2, SD = 1.41 vs home = 38.7, SD = 1.85, *p* = .392). Of those 12, only one was from the comparison group. Of the remaining 11 infants from the methadone group, *N* = 11 (100%) were infants who received pharmacotherapy treatment for NAS. Although there were no systematic differences across summary scores, there were three summary scale scores where there were significant differences. More Stress Abstinence (M = .15, SD = .11, vs M = .21, SD = .11, *p* = .024) and Arousal (M = 4.09, SD = .75 vs M = 4.68, SD = .65, *p* < .017) were observed among infants administered at home, and scores on Quality of Movement that were more optimal (M = 4.30, SD = .87 vs M = 3.23, SD = .71, *p* = < .001).

Table 4 describes the neurobehavior of infants born to mothers maintained on methadone during pregnancy and unexposed comparison infants based on the GLIM analyses. Results given include the unadjusted and adjusted means, the unstandardized regression coefficients (B), and the Wald 95% confidence intervals (CI) adjusted for the covariates for the 12 NNNS summary scores and the seven subcategories of abstinence.

**Table 4:** NNNS summary scores by exposure to methadone maintenance treatment unadjusted and adjusted for covariates.

NNNS Summary Scores Mean (SD)		Unadjusted			Adjusted		Unadj	Adj	B	Sig
	*N*	Methadone	*N*	Comparison	Methadone	Comparison	*P*	*P*	(Wald 95% C-I)	Cov
Attention	71	5.83 (1.54)	98	6.78 (1.23)	5.83 (1.54)	6.78 (1.42)	**< .001**	**< .001**	-0.95 (-1.38 - -0.52)	
Arousal	85	4.17 (0.79)	103	3.78 (0.56)	4.17 (0.66)	3.77 (0.81)	**< .001**	**< .001**	0.40 (0.21–0.60)	4
Regulation	85	5.39 (0.97)	103	6.22 (0.80)	5.38 (0.88)	6.23 (0.79)	**< .001**	**< .001**	-0.86 (-1.16 - -0.59)	2,5
Handling	84	0.30 (0.37)	103	0.18 (0.36)	0.28 (0.37)	0.42 (0.31)	**.009**	.094	0.07 (-0.1 - .14)	4
Quality of Movement	84	4.16 (0.92)	103	4.87 (0.71)	4.15 (0.92)	4.87 (0.71)	**< .001**	**< .001**	-0.16 (-0.22–0.11)	4
Excitability	86	3.60 (2.69)	103	1.90 (1.93)	3.67 (1.95)	1.85 (1.42)	**< .001**	**< .001**	0.50 (0.32–0.67)	2
Lethargy	86	3.29 (2.37)	103	3.06 (1.43)	3.29 (2.37)	3.06 (1.43)	.424	.424	0.23 (-0.34–0.80)	
Non-Optimal Reflex	86	4.02 (1.95)	103	3.15 (1.93)	4.02 (1.95)	3.15 (1.93)	**.003**	**.003**	0.88 (0.30–1.46)	
Asymmetrical Reflex	86	0.72 (1.06)	103	0.56 (0.78)	0.72 (1.06)	0.56 (0.78)	.241	.241	0.10 (-0.06–0.26)	
Hypertonicity	86	0.51 (0.65)	103	0.08 (0.31)	0.49 (0.58)	0.10 (0.53)	**< .001**	**< .001**	0.34 (0.21–0.44)	1
Hypotonicity	86	0.15 (0.37)	103	0.17 (0.41)	0.15 (0.37)	0.17 (0.41)	.692	.692	0.02 (0.05 - -0.12)	
Stress Abstinence	86	0.16 (0.07)	103	0.09 (0.07)	0.15 (0.07)	0.09 (0.07)	**< .001**	**< .001**	0.05 (0.03–0.07)	1,2,3
Physiological	86	0.10 (0.21)	103	0.05 (0.20)	0.10 (0.21)	0.05 (0.20)	.149	.149	0.04 (-0.02–0.10)	
Autonomic	86	0.24 (0.20)	103	0.19 (0.18)			.091	**-**	0.04 (-0.01–0.08)	
CNS	86	0.21 (0.18)	103	0.12 (0.20)	0.21 (0.15)	0.11 (0.15)	**< .001**	**< .001**	0.08 (0.04–0.12)	4
Skin	86	0.11 (0.10)	103	0.08 (0.10)			.061	-	0.03 (0–0.06)	
Visual	82	0.11 (0.09)	103	0.07 (0.08)	0.1 1(0.09)	0.07 (0.08)	**.002**	**.002**	0.04 (0.01–0.06)	
GI	86	0.05 (0.10)	103	0.02 (0.10)	0.05 (0.10)	0.02 (0.10)	**.051**	**.051**	0.03 (0–0.06)	
State	86	5.61 (1.04)	103	5.24 (1.01)	5.61 (1.04)	5.24 (1.04)	**< .001**	**< .001**	0.13 (0.09–0.16)	

Significant covariates in the models of NNNS summary scores: 1 = Ritalin, 2 = Benzodiazepines, 3 = Infant birth weight, 4 = Corrected gestational age NNNS completed, 5 = gestational age at birth (completed weeks).

Relative to the comparison group, MMT exposed infants had a number of summary scores on the NNNS that suggest poorer neurobehaviour in a number of domains. After adjustment for covariates MMT exposed infants had poorer attention (M = 5.83, SD = 1.54 vs M = 6.78, SD = 1.42, *p* < .001), regulation (M = 5.38, SD = 0.88 vs M = 6.23, SD = 0.79, *p* = < .001), and quality of movement (M = 4.15, SD = 0.92 vs M = 4.87, SD = 0.71, *p* = < .001). They were also significantly more excitable (M = 3.67, SD = 1.95 vs M = 1.85, SD = 1.42, *p* = < .001), more easily aroused M = 4.17, SD = 0.66 vs M = 3.77, SD = 0.81, *p* = < .001), exhibited more non-optimal reflexes (M = 4.02, SD = 1.95 vs M = 3.15, SD = 1.93, *p* = .003), and more hypertonicity (M = 0.49, SD = 0.58 vs M = 0.10, SD = 0.53, *p* = < .001). Finally, MMT exposed infants exhibited more total stress abstinence signs (M = 0.15, SD = 0.07 vs M = 0.09, SD = 0.07, *p* = < .001) and higher scores than comparison infants on CNS (M = 0.21, SD = 0.11 vs M = 0.21–0.11, SD = 0.15, *p* = < .001), visual (M = 0.11, SD = 0.09 vs M = 0.07, SD = 0.07, SD = 0.08, *p* = .002), genitourinary (GI) (M = 0.05, 0.10, SD = 0.10 vs M = 0.05, SD = 0.10, *p* = .051) and state (M = 5.61, SD = 1.01 vs M = 5.24, SD = 1.01, *p* < .001) indices of abstinence.

The effect of maternal methadone dose across pregnancy on infant neurobehavioral scores was examined using GLIM before and after adjustment for the effects of covariates. Of interest was whether high maternal methadone dose (≥65mg) was associated with greater neurobehavioral decrements in comparison to infants born to low (≤64mg) methadone dose and no dose (control) mothers. Compared to the low and no dose groups (M = 0.38, SD = 0.58, M = 0.07, SD = 0.06, respectively) the high dose group had higher scores on hypertonicity (M = 0.64, SD = 0.09, B = -0.56, CI -0.35 - -0.76) after adjusting for covariates. No effects of dose were associated with any other NNNS summary scores.

### NNNS profiles

The optimal number of profiles were determined by fitting two to five profiles. The decrease in the BIC value adjusted for sample size with each increase in profile suggested improved goodness of fit (see Table 2). The five-profile model had the smallest BIC, but the fifth group contained only 5 infants suggesting that a four-profile model was the best fit for the data. Model fit and the optimum number of profiles were further evaluated with the bootstrap log likelihood ratio (LR) difference test [59], and the average posterior probability (entropy). Both tests supported a four-profile model. To determine whether the four-profile solution specified four unique classes, NNNS summary scores were compared across profiles and found to be significant for all scales (Table 5).

**Table 5:** NICU Network Neurobehavioral Scale summary scores by profiles.

NNNS Summary Score	Profile 1 *N* = 31	Profile 2 *N* = 84	Profile 3 *N* = 50	Profile 4 *N* = 24	*P* value
Attention	4.53 (1.04)	7.22 (0.78)	6.46 (1.12)	5.14 (1.66)	< .001
Handling	0.21 (0.27)	0.11 (0.20)	0.33 (0.33)	0.46 (0.38)	< .001
Self Regulation	5.52 (0.55)	6.61 (0.61)	5.45 (0.56)	4.42 (0.73)	< .001
Arousal	3.39 (0.46)	3.58 (0.34)	4.41 (0.44)	5.00 (0.44)	< .001
Excitability	0.39 (0.80)	0.31 (0.56)	1.84 (1.42)	3.79 (1.50)	< .001
Lethargy	5.71 (1.35)	2.95 (1.19)	2.20 (1.26)	2.63 (2.81)	< .001
Hypertonicity	13 (42%)	3 (4%)	8 (16%)	13 (54%)	< .001
Hypotonicity	9 (29%)	7 (8%)	8 (16%)	4 (17%)	.049
Non-optimal Reflexes	4.29 (1.64)	2.95 (1.73)	3.42 (1.85)	4.92 (2.83)	< .001
Asymmetric Reflexes	0.58 (0.76)	0.44 (0.66)	0.66 (0.77)	1.33 (1.61)	< .001
Quality of Movement	4.51 (0.75)	4.98 (0.63)	4.50 (0.73)	3.20 (0.78)	< .001
Stress Abstinence	0.58 (0.76)	0.44 (0.66)	0.66 (0.77)	1.33 (1.60)	< .001

Mean (SD) and Number (%).

**Fig 1:**
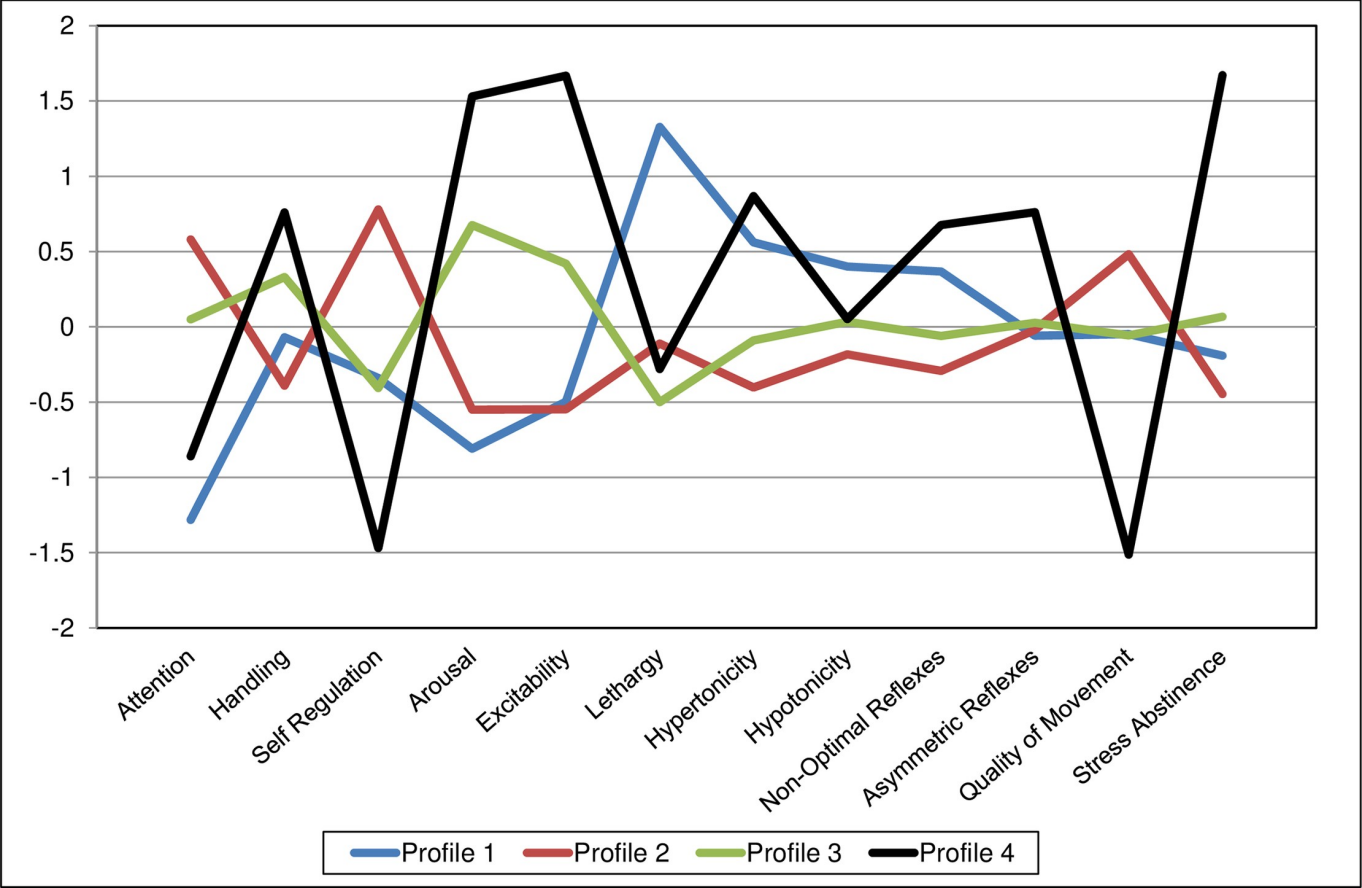
Standardized NNNS Scales plotted by profile.

Standardized summary scores were plotted to demonstrate the pattern of each profile (Fig 1). Profile 1 included 31 (18%) infants. This profile was distinguished by the lowest attention and arousal and the highest lethargy scores, but with scores close to average on handling, self-regulation, asymmetric reflexes, quality of movement, and stress abstinence (Table 5). The 84 (44%) infants in profile 2 showed the most optimal neurobehavioral pattern requiring the least amount of handling, with better attention and self-regulation, and lower levels of arousability than profiles 3 and 4. They also demonstrated average levels of lethargy, the lowest scores on hypertonicity, hypotonicity, non-optimal reflexes and stress abstinence, and the highest quality of movements score. The 50 (27%) infants in profile 3 scored near the average on all measures, but had higher scores on excitability and arousal than profiles 1 and 2. Profile 4 infants showed the poorest profile of neurobehaviour. These 24 (13%) infants had poorer attention scores than profiles 2 and 3, required the most handling, had the lowest scores on self-regulation, and the highest on arousal, excitability, hypertonicity, non-optimal and asymmetric reflexes, and exhibited the highest stress abstinence and the poorest quality of movement.

#### Validation and prediction of NNNS profiles

To evaluate the profile classifications, profiles 1, 2 and 3 were compared to profile 4 across a range of maternal and infant characteristics (Table 6). Infants in profile 4 were significantly more likely to be born to a mother on government welfare (83% vs 47%, p = .001), and with a history of significantly more pregnancies and terminations than those in the other 3 profiles. Profile 4 infants had significantly more prenatal exposure to legal and illegal psychoactive drugs. Mothers of these infants were receiving MMT in all 3 trimesters and used tobacco and stimulants during pregnancy.

**Table 6:** Comparison of profile 4 vs profiles 1–3.

	Profile 4 vs Profiles 1–3		
	Profile 4 *N* = 24	Profiles 1–3 *N* = 165	*P* value
**Demographics**			
Maternal age (yr)	30.13 (4.70)	30.68 (6.64)	.691
Maori	5 (21%)	23 (14%)	.389
NZ European	17 (71%)	124 (76%)	.578
Other European	3 (13%)	14 (9%)	.534
Pacific islands, Asian & Other	1 (4%)	9 (6%)	.783
Single or no partner at birth	8 (33%)	44 (27%)	.494
No educational qualification	14 (58%)	76 (46%)	.261
Receiving government benefit	20 (83%)	77 (47%)	**.001**
**Prenatal Legal or Illegal Psychoactive Drug Exposure**			
Receiving MMT	21 (88%)	76 (46%)	**< .001**
Receiving MMT all 3 trimesters	20 (83%)	66 (41%)	**< .001**
Alcohol	4 (17%)	32 (19%)	.751
Tobacco	21 (88%)	76 (46%)	**< .001**
Marijuana	8 (33%)	76 (46%)	.304
Opiates other than MMT	5 (21%)	17 (10%)	.133
Stimulants	7 (29%)	10 (6%)	**< .001**
Benzodiazepines	4 (17%)	19 (12%)	.471
Prescribed medication	8 (33%)	33 (20%)	.139
Used more than 2 psychoactive drugs in pregnancy	9 (38%)	28 (17%)	**.018**
**Maternal Obstetric History**			
Primigravida	3 (13%)	42 (26%)	.164
History of Termination	11 (46%)	37 (22%)	**.014**
History of still born or miscarriage	11 (46%)	51 (31%)	.146
Number of live births	1.42 (1.21)	1.36 (1.50)	.854
Number of previous pregnancies	3.63 (3.16)	2.33 (2.48)	**.023**
Any maternal mental illness	11 (46%)	58 (35%)	.310
**Infant Clinical Outcomes**			
Male	17 (71%)	79 (48%)	**.036**
Gestational age (wk)	38.07 (2.06)	39.16 (1.67)	**.004**
Born Preterm (< 37 wk)	5 (21%)	13 (8%)	**.043**
Birth weight (gm)	2973.33 (412.45)	3297 (572.40)	**.008**
Birth head circumference (cm)	33.65 (1.65)	34.44 (1.50)	**.017**
Birth length (cm)	49.67 (3.12)	51.49 (3.23)	**.010**
> 2 weeks in special care nursery	10 (42%)	22 (14%)	**.001**
> 2 weeks total stay in hospital	8 (33%)	19 (12%)	**.005**
NAS treated with morphine	19 (83%)	54 (33%)	**< .001**
> 1 month morphine treatment	18 (78%)	42 (27%)	**< .001**
NAS treated with phenobarbital	5 (22%)	13 (18%)	**.038**
> 1 month phenobarbital treatment	5 (23%)	9 (6%)	**.005**
**Development at 24 Months**			
BAYLEY-II MDI	78.96 (18.61)	87.94 (18.00)	**.025**
BAYLEY-II PDI	80.92 (24.05)	92.03 (17.81)	**.007**

*N* (%) or Mean (SD).

Given that infants in profile 4 showed the poorest performance on the NNNS, we hypothesized that this group would have poorer clinical outcomes at birth and poorer neurodevelopment at age 2 years. As shown in Table 6, a significantly higher percentage of infants in profile 4 were male (71% vs 48%, *p* = .036), born preterm (21% vs 8%, *p* = .043), and were significantly smaller at birth. A higher proportion of profile 4 infants spent more than 2 weeks in a special care baby unit (42% vs 14%, *p* = .001) and more than 2 weeks in hospital postnatally (33% vs 12%, *p* = .005). Finally, a higher percentage of these infants required pharmacological (morphine) treatment for NAS (83% vs 33%, *p* = < .001) typically for longer than a month (78% vs 27%, *p* = < .001). At 24 months, profile 4 infants had significantly lower mean scores than the other three profiles on the BSID-II, MDI (M = 78.96, SD = 18.61 vs 87.94, SD = 18.00, *p* = .025, partial *η*^2^ = .03) and PDI (M = 80.92, SD = 24.05 vs M = 92.03, SD = 17.81, *p* = .007, partial *η*^2^ = .04).

## Discussion

This prospective, longitudinal study compared the neurobehavior of infants born to mothers receiving MMT for opioid dependence with a group of non-methadone exposed infants born to mothers who were randomly selected from the general obstetric population at the largest maternity hospital in the region. Using the summary scores from the NNNS and a clustering technique using all data, we characterized four distinct profiles of infant neurobehavior. These profiles were associated with differing clinical outcomes at birth and neurodevelopment at age 24 months.

The main outcome of this study was the identification of unique effects of MMT on newborn neurobehavior. After controlling for covariates, including other psychoactive drug use and socioeconomic status, MMT exposed infants had poorer attention, were more easily aroused, more excitable, and had poorer behavioral regulation than unexposed infants. They also exhibited poorer quality of movement, non-optimal reflexes, and more hypertonicity. Finally, they exhibited more signs of stress abstinence that spanned disturbances to state regulation and the CNS, visual and genitourinary systems. An examination of the dose effects on these patterns of behavior found that increased hypertonicity alone was associated with exposure to higher maternal methadone doses (>65 mg per day).

Of the four discrete neurobehavioral profiles identified, the poorest neurobehavioral functioning was observed in the small subsample of 24 (13%) infants in profile 4. These infants exhibited the most stress abstinence, poorest state regulation, and ability to maintain an alert attentive state; were more easily aroused and excitable; required more handling/soothing, were more hypertonic and showed more non-optimal and asymmetric reflexes. This group was over-represented by MMT exposed infants (88%) and a higher proportion were exposed prenatally to tobacco (88%) and stimulants (29%). Their mothers had more pregnancies and terminations and were almost twice as likely to have been in MMT throughout their pregnancy relative to Profile 1–3 infants. Profile 4 infants also exhibited more worrisome clinical outcomes at birth, including being more likely to be born preterm (21%) and symmetrically smaller than infants in profiles 1–3. In addition, a higher proportion (78%) required extended (>1 month) NAS treatment (83%) and spent longer in hospital (33%). At the corrected age of 24 months, these infants were more likely to be lagging behind their peers neurodevelopmentally, obtaining mean cognitive and psychomotor scores that were 1.5 and > 1 standard deviation below the norms, respectively. In contrast, group 2 had the most optimal neurobehavioral profile and tended to be over-representative of control and/or lower dose MMT mothers (Table 5).

The finding that MMT exposed infants have a more dysregulated pattern of neurobehavior at birth than unexposed infants is consistent with the work of Velez et al. [31] and to some extent Heller et al. [32] as reviewed earlier. However, in contrast to Heller et al. [32] who found that adverse neurobehavioral findings were confined primarily to NAS treated infants, we found MMT exposed infants exhibited more dysregulated neurobehavior, regardless of whether they required treatment for NAS or not. The possible absence of further associations between MMT and poorer neurobehavioral outcomes by Heller et al. [32] may reflect limited statistical power due to their small sample size. It may also, as the authors suggest, reflect maturation or developmental changes in the CNS over the first month. Coyle et al. [29] found changes over the first month postnatally in NNNS summary scores in both MMT exposed and buprenorphine exposed infants that showed more optimal neurobehavioral function with increasing time since birth. Further research examining the effects of prenatal opioid exposure on the developing brain, not just at birth but over the longer term, will be important in understanding the potential neuropathological mechanisms underlying these early neurobehavioral observations.

Our findings that a discrete profile of neurobehavior could be identified that showed the most extreme negative scores on the NNNS and was associated with poorer clinical and neurodevelopmental outcomes later in infancy is consistent with the findings of Liu et al. [51]. They found the most negative pattern of neurobehavior was associated with a group of children who were prenatally exposed to cocaine plus opiates, tobacco and marijuana. Children in this group compared to the other profiles were more likely to be born preterm, weigh less at birth, and more likely to have an abnormal ultrasound reading at 1 month. Over the first 4 years of life, nearly 40% had clinically significant behavior and school readiness problems, and approximately 35% had a low IQ.

These findings highlight a unique profile of children that appear to be at particularly high neurodevelopmental risk. They also suggest the possibility of early detection of these infants prior to hospital discharge using existing medical and family information alongside systematic and careful neurobehavioral evaluation of the infant. Detecting specific neurobehavioral deficits early in the postnatal period that are associated with poorer neurodevelopmental outcomes later in childhood could also inform the development of intervention programs targeted to those exhibiting these deficits. Finally, these data do raise serious concerns about the longer-term health and neurodevelopment of these children, especially since many are likely to be discharged into challenging home circumstances characterized by social adversity, maternal mental illness and ongoing parental psychoactive drug use [60]. Thus, further longer term follow-up studies will also be important, both in terms of assessing the predictive utility of neurobehavioral observations and clarifying the longer-term developmental and psychosocial needs of these children and their families [61].
